# Early Postpartum Sonographic Assessment of the Uterotomy After Successful Vaginal Birth After Caesarean Section: An Observational Cohort Study

**DOI:** 10.3390/medicina62091669

**Published:** 2026-08-31

**Authors:** Marie Christine Pohle-Rüffer, Tilman Born, Luisa Hirluksch, Maurice Kappelmeyer, Maximilian Rauh, Angela Köninger

**Affiliations:** 1 Department of Gynecology and Obstetrics, Hospital St. Hedwig of the Order of St. John, University of Regensburg, Steinmetzstrasse 1-3, 93049 Regensburg, Germany; 2 Laboratory of Translational Perinatology, University of Regensburg, Am Biopark 9, 93053 Regensburg, Germany

**Keywords:** vaginal birth after caesarean section, trial of labor after caesarean section, uterine niche, caesarean section, transvaginal sonography, uterine scar, lower uterine segment

## Abstract

*Background and Objectives*: The rising rate of caesarean sections worldwide has led to an increasing number of women facing the choice between a trial of labor after caesarean section and elective repeat caesarean delivery. A uterine rupture is the most serious complication of trial of labor after caesarean section and, from a medical point of view, the main reason for an elective repeat caesarean section. Previous studies on the predictive value of lower uterine segment thickness have shown inconsistent results. Conversely, it is unclear what characteristics of the uterotomy site allow for an uncomplicated vaginal birth, even in the presence of defective healing. This study aimed to evaluate the sonographic features of the lower uterine segment in the early postpartum period after successful vaginal birth after caesarean section. *Materials and Methods*: We conducted a single-center observational cohort study including 30 women after vaginal birth after caesarean section. Transabdominal and transvaginal sonography were performed within the first days postpartum. Measurements included residual myometrial thickness, defect dimensions, and healing ratio after delivery to perform an ex-post analysis with regard to successful vaginal birth. Descriptive statistics were applied. *Results*: Transabdominal ultrasound (TAS) visualized the uterotomy in 90.0% of patients (27/30), with a median residual myometrial thickness of 12.0 mm (interquartile range 8.0–19.0). Among these, a uterine defect ≥ 2 mm was present in 63.0% (17/27 of visualized cases; 56.7% [17/30] of the entire cohort). Transvaginal ultrasound (TVS) visualized the uterotomy in all patients (29/29), with a median residual myometrial thickness of 9.9 mm (interquartile range 7.5–14.5); a defect was present in 82.8% (24/29 of visualized cases; 80.0% [24/30] of the entire cohort), with a median depth of 9.9 mm (interquartile range 6.5–15.6). *Conclusions*: Early postpartum sonographic evaluation of the uterotomy scar after vaginal birth after caesarean section frequently reveals even large sonographic uterine wall defects. This study design is not suitable for predicting uncomplicated vaginal delivery, but it demonstrates for the first time—on a retrospective basis—that even large defects in the anterior uterine wall are compatible with an uncomplicated vaginal birth after caesarean section.

## 1. Introduction

As caesarean section (CS) rates continue to rise worldwide, an increasing number of women are faced with the decision between attempted vaginal delivery (TOLAC, trial of labor after caesarean delivery) and elective repeat caesarean delivery (ERCD) in a subsequent pregnancy [1,2]. Although vaginal birth after caesarean section (VBAC) is not associated with increased rates of PPH (peripartum hemorrhage), hysterectomy, maternal blood transfusion, postpartum infection, or neonatal admission to a neonatal intensive care unit [3,4], there has been an increasing trend towards ERCD, with fewer women attempting VBAC in recent decades [5].

A rare but potentially dangerous complication of TOLAC is uterine rupture, which is associated with high maternal and neonatal morbidity and mortality [6,7,8,9,10]. While a catastrophic uterine rupture can almost always be avoided from a medical standpoint through ERCD, other risks increase. In the context of repeat caesarean sections, the placenta accreta spectrum (PAS) is particularly noteworthy, involving maternal risks ranging from hysterectomy to loss of fertility [11], as well as neonatal risks such as preterm birth, respiratory distress syndrome, and the need for intensive care [12].

In summary, the decision between ERCD and TOLAC presents a major challenge, and new insights into the safety of TOLAC are of the utmost relevance.

TOLAC is successful in 60–85% of cases [13,14,15,16]. The success rate of TOLAC increases with each subsequent successful VBAC [17,18], and a number of predictive models have been available for many years to guide decisions about the mode of delivery after CS. One of the first available prediction models was that of Grobman et al. [19]. The most commonly considered factors are maternal age and maternal BMI (body mass index), previous vaginal deliveries and previous CS as well as their indication, fetal birth weight, gestational age, and Bishop score [20,21,22]. Another factor of major importance in determining the likelihood of TOLAC success is wound healing of the uterotomy from the previous CS. Several studies have investigated the relationship between the thickness of the lower uterine segment (LUS) or the residual myometrium (RMT) and the probability of uterine rupture or dehiscence; however, the results so far are controversial [15,16,23,24,25,26].

The presence of a uterine niche is important in assessing wound healing after CS. Niches occur in more than 50% of patients after CS and are defined as an indentation of the myometrium in the area of the uterotomy with a depth of at least 2 mm, observed by transvaginal ultrasound in the non-pregnant state [1,27]. The risk of uterine rupture during TOLAC in subsequent pregnancies is increased, particularly in the presence of a large scar defect [6].

Definitions of large uterine niche defects vary across studies. Most commonly, a pre-pregnant residual myometrial thickness (RMT) of less than 3 mm is considered indicative of a large defect [28,29]; alternatively, some authors have proposed a healing ratio (HR, the ratio between residual and adjacent myometrium) below 0.5 as a threshold for defining large defects [30].

The most commonly used methods for assessing wound healing after uterotomy are transvaginal sonography (TVS) and hysterosonography (HSG), with HSG considered one of the most sensitive modalities [30,31].

Given the high success rate of VBAC and, at the same time, the high probability of a uterine defect after CS, the question arises as to what size of scar defect still allows for VBAC and which patients can be encouraged to undergo TOLAC.

This study aimed to characterize the sonographic appearance of the lower uterine segment in women following a successful VBAC in the early postpartum period, with particular focus on RMT, defect dimensions, and the HR as markers of uterine scar morphology. By providing descriptive data on these sonographic findings, this study aims to contribute to a better understanding of uterine scar dimensions that allow successful VBAC and to generate hypotheses for future research on their potential clinical relevance.

## 2. Materials and Methods

For this observational cohort study, conducted at the Department of Gynecology and Obstetrics at the University Clinic St. Hedwig of the Order of St. John, Regensburg, Germany, postpartum patients were collected prospectively between March 2024 and August 2024. The methodological procedures of this study were reviewed and approved by the Ethics Committee of the University of Regensburg, Germany (23-3409-101).

### Patient Selection

This study was designed to analyse anatomical parameters of the lower uterine segment (LUS) that show morphological scar integrity, reflected by a measurable defect/indentation within the uterine incision site after a CS and a subsequent successful vaginal birth. The aim of the study was not to explore in a prospective way the success probability of TOLAC with regard to a defect but to observe the uterotomy integrity ex post and therefore retrospectively after successful TOLAC.

Due to the massive stretching of the uterus during the third trimester and during labor, it appears impossible to measure the exact dimensions of a uterine wall defect at that time. To obtain the most realistic comparison possible between the wall thickness of the adjacent myometrium and the incised uterine tissue, a rational approach is hypothesized to examine the symmetrically contracted uterus in the regions of interest immediately postpartum. Provided no uterine rupture and no repeated incision occur, postpartum ultrasound findings are assumed to approximate best the myometrial relationship within and outside the uterotomy site as presented at the time of delivery, although this remains hypothetical.

Consecutively, patients who had a successful VBAC were invited to participate in the study during their hospital stay within the first six days after delivery. A control group of women with failed TOLAC was not included ex post since any kind of ERCD makes it impossible to measure the uterotomy site as it was prior to delivery.

The inclusion criterion was a successful VBAC after at least one previous CS. After receiving detailed information about the aims, procedures, and possible side effects of the study, all participants gave written informed consent before the start of the examination. During the study period, all eligible patients were consecutively invited to participate with the exception of very few women with relevant language barriers.

The study protocol included a standardized medical history (age and BMI) at the time of examination, including medications, pre-existing medical conditions, previous surgery, and pre-pregnancy symptoms associated with caesarean scar disorder (menstrual irregularities, dysmenorrhea, hypermenorrhea, postmenstrual spotting, pelvic pain, secondary infertility) [1].

**Figure 1:**
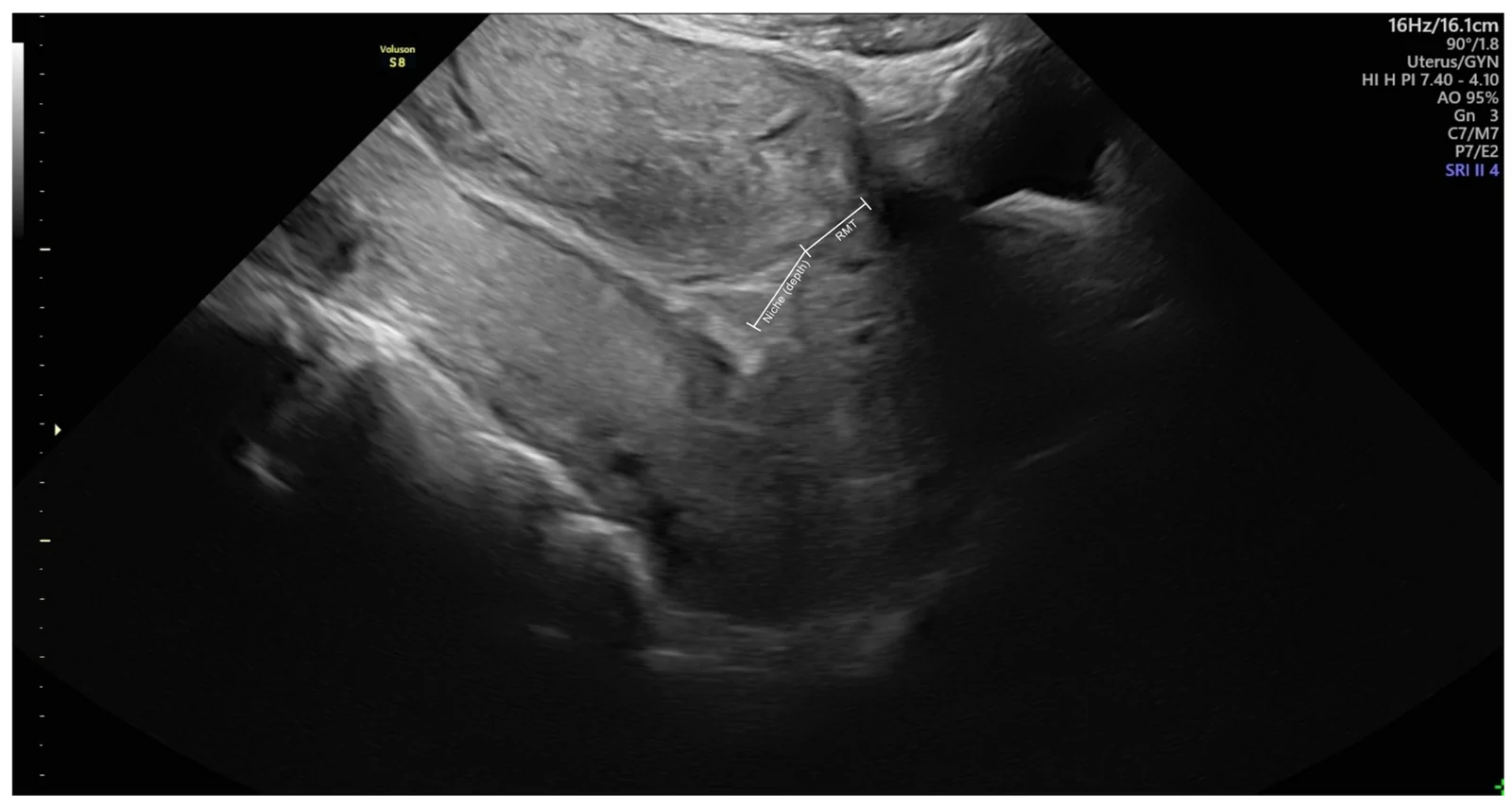
Uterus of a patient in sagittal section using transabdominal sonography (TAS). The lower uterine segment with a uterine defect after vaginal birth after caesarean section (VBAC) can be seen.

**Figure 2:**
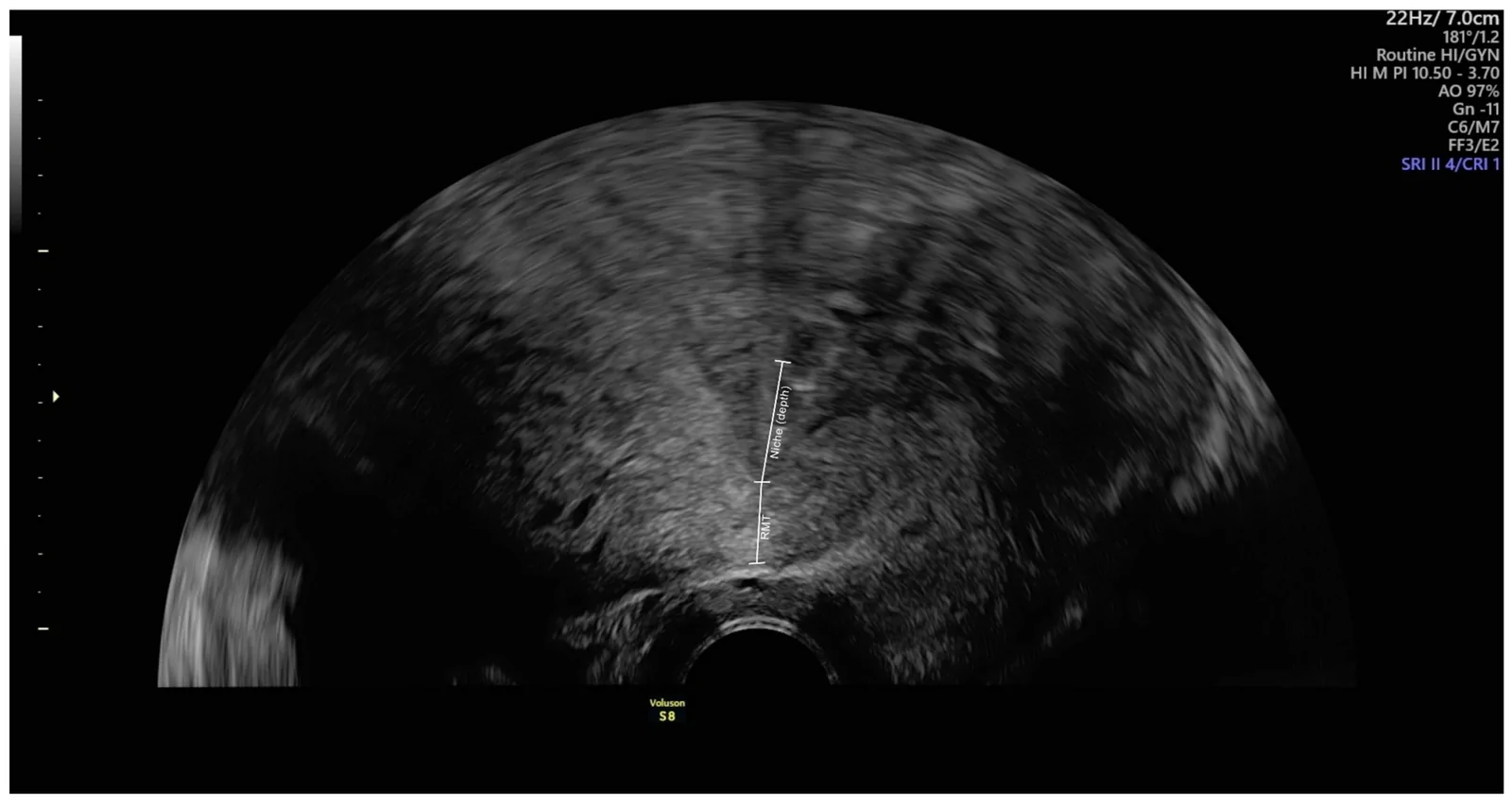
Uterus of a patient in sagittal section using transvaginal sonography (TVS). The lower uterine segment with a uterine defect after vaginal birth after caesarean section (VBAC) can be seen.

Study participants were examined using both Grayscale B-mode image TAS and TVS; examples are shown in Figure 1 and Figure 2, respectively. We focused on examination of the lower uterine segment, including visualization of the uterotomy and, if present, a uterine defect. As the examination was performed shortly after birth and women had lochia, the use of contrast medium was not indicated. By default, color Doppler is used as a supplementary tool when structures are unclear and the vascular supply raises questions.

In addition, sonographic assessment for retained products of conception was performed in all patients.

All examinations were performed using a high-resolution ultrasound machine (GE [General Electric] Voluson S8) and ViewPoint™ 6 software (GE Healthcare) for data collection. Transabdominal sonography was performed using a curved-array abdominal transducer (4C-RS, 2.0–5.0 MHz), and transvaginal sonography was performed using a high-frequency endocavitary transducer (E8C-RS, 4.0–10.0 MHz). Both TAS and TVS were performed with the patient in a supine position, without a defined bladder filling protocol as the postpartum uterus is markedly enlarged and typically provides an adequate acoustic window without a full bladder.

All examinations were performed jointly by two investigators throughout the study period including a highly experienced physician (DEGUM level I certification in obstetric ultrasound)—following a four-eyes principle, whereby both examiners were present at each examination and all measurements were obtained in consensus. Instruction and final supervision was performed by a senior consultant (level DEGUM II for gynecology and obstetrics).

All measurements were obtained in the midsagittal plane, with additional assessment of defect width and RMT in the transverse plane, as described above.

Examinations were performed between the first and sixth postpartum day.

Diagnosis and definition of a caesarean scar defect: In the case of defect, any visible indentation at the uterotomy site of at least 2 mm, the indentation was visualized in all planes according to standardized guidelines, and the exact size of its extension was measured according to Jordans’ criteria for assessment of niches [27]. This included defect length, depth, and residual myometrial thickness (RMT) in the sagittal plane, as well as defect width and RMT in the transverse plane. It should be noted that Jordans’ criteria—including the 2 mm depth threshold used to define the presence of a defect—were originally developed and validated for defect assessment in the non-pregnant uterus, after at least three menstrual cycles postpartum, and their applicability to the early postpartum period (1–6 days after delivery) has not been established. Therefore, we used the parameters described by Jordans et al. [27] and report RMT, defect depth, and defect frequency descriptively alongside the healing ratio (HR), which represents the ratio between the residual and the total myometrial thickness. Among these parameters, we consider the HR the most informative for this early postpartum setting, as it expresses a relative rather than an absolute measure of defect depth. To avoid confusion with the established definition of a defect in the non-pregnant state, we consistently use the term “defect”.

HR is defined as the RMT divided by the sum of the RMT and the defect depth in the sagittal plane. This purely morphological sonographic parameter only reflects an anatomical structure, but no biomechanical scar strength in general.

Patient demographics and ultrasound measurements were collected, and descriptive statistics were calculated for the following parameters: time after CS, age, BMI, gestational age at CS, sagittal RMT, defect depth, and healing ratio. All statistical analyses were performed using SigmaPlot (version 14.0, Systat Software, Inc., San Jose, CA, USA).

## 3. Results

In total, 30 patients were included in the study; 29 received both TAS and TVS, while in one patient TVS was not possible due to pain. The mean age of the patients at the time of examination was 32.5 ± 5.3 years, with a mean BMI of 29.4 ± 5.8 kg/m^2^. Eighty percent of patients (24/30) had one CS in their medical history, and 20% (6/30) also had vaginal deliveries, five of which were VBAC. Regarding the type of the CS, 30% of patients (9/30) had undergone elective CS, 60% of patients (18/30) an unplanned CS, while in 10% of patients (3/30) this information was not available. The mean interval between CS and vaginal delivery was 57.8 ± 36.1 months (IQR 31.7–71.9). In addition, two patients had previously undergone an abortion curettage, and one patient had undergone hysteroscopic septal ablation of a uterine septum. No patient showed sonographic evidence of retained products of conception requiring therapeutic intervention. The demographic and sonographic data are shown in Table 1.

Using TAS, the uterotomy could be visualized in 90.0% of patients (27/30; 95% CI 74.4–96.5%). The median RMT in these patients was 12.0 mm (IQR 8.0–19.0). Among these 27 patients, a uterotomy defect of at least 2 mm, within the area of the uterotomy, was found in 63.0% (17/27; 95% CI 44.2–78.5%), corresponding to 56.7% (17/30) of the entire cohort, with a median depth of 12.2 mm (IQR 7.7–14.8) and a median HR of 0.55 (IQR 0.30–0.62).

Using TVS, the uterotomy could be visualized in all examined patients (29/29, 100%; 95% CI 88.3–100%). The median RMT was 9.9 mm (IQR 7.5–14.5). A uterotomy defect of ≥2 mm was detected in 82.8% (24/29; 95% CI 65.5–92.4%) of visualized cases, corresponding to 80.0% (24/30) of the entire cohort, with a median defect depth of 9.9 mm (IQR 6.5–15.6). The median healing ratio was 0.50 (IQR 0.33–0.66).

## 4. Discussion

This observational cohort study aimed to characterize the sonographic appearance of the LUS in women after successful VBAC during the early postpartum period, describing the relationship between the uterotomy region and the adjacent myometrium as it presents in the immediate postpartum period. We can only hypothesize that these findings most likely correspond to the intrapartum condition as well.

It was possible to visualize the uterotomy and determine the RMT between the first and sixth day postpartum in 100% (29/29) of patients using TVS. The median RMT was 9.9 mm (IQR 7.5–14.5). In 82.8% of these participants (24/29), a uterine defect with a median depth of 9.9 mm (IQR 6.5–15.6) could also be detected. Measured against the thickness of the total myometrium, this corresponds to 49%; correspondingly, the healing ratio was 0.51 on average (median 0.50, IQR 0.33–0.66).

The detection rate for defects was evidently higher with TVS than with TAS. This is consistent with everyday clinical experience in gynecological ultrasound. Although the small size of our cohort does not allow for a formal paired agreement analysis between TAS and TVS, the higher detection rate observed for TVS in the present cohort is consistent with the well-documented lower sensitivity of TAS for identifying uterine niches and residual myometrial defects [32]. Based on this, TVS may be preferable when both modalities are feasible; however, data are not sufficiently suitable to support a definitive, generalizable recommendation, and paired agreement analysis in future, larger studies is recommended. The subjects reported no pain when a very narrow ultrasound probe was used. Nevertheless, TVS should be used with caution during the first postpartum week; in this study, it was employed specifically to achieve the best possible resolution.

In summary, only half of the participants with a successful VBAC had an intact myometrial wall of at least 50%, and in a quarter of participants, 30% or less of the myometrium was still intact. The minimum observed RMT was 5.6 mm, and the minimum HR was 0.25. The correlation between wound healing of the LUS and the probability of TOLAC success has been recognized for decades [33]. In particular, studies have aimed to stratify the risk of TOLAC by determining the thickness of the LUS. Since 1996, there have been attempts to define a clear cut-off value for the LUS above which TOLAC appears promising and is associated with a low risk of uterine rupture.

In 2022, for example, a Chinese study involving more than 700 women showed that the sonographically measured thickness of the LUS is associated with the probability of a successful TOLAC. The LUS was measured using TAS before the start of TOLAC or CS. At 22 ± 11 mm, the thickness of the LUS was significantly greater in patients who successfully underwent VBAC than in patients with failed TOLAC, in whom the thickness of the LUS was 17 ± 10 mm (*p* < 0.001) [15]. The overall high measured thickness of the LUS in the third trimester in this study is notable. It is known that the reproducibility of measurements of the lower uterine segment or the RMT is limited in advanced pregnancy [34]. Nevertheless, these findings are consistent with the hypothesis that uterine scar healing is associated with the probability of successful TOLAC. In the present study group, the RMT determined by TAS postpartum was substantially lower, at only 12.0 mm (IQR 8.0–19.0). It should be noted that in our cohort, the measurement was carried out only when the uterotomy could be reliably visualized. Another prospective cohort study including 236 women after at least one CS described a cut-off value for the thickness of the prepartum LUS of <2.3 mm for an increased probability of uterine rupture during TOLAC. Measurements were carried out between 36 and 39 weeks of gestation using TAS and, if necessary, TVS. The median thickness of the LUS was 2.8 mm. The difference in mean LUS thickness between the above-mentioned studies is striking and again raises the question of the reproducibility of LUS thickness measurements on the pregnant uterus in advanced weeks of pregnancy [23].

As part of their study on the predictive power of RMT for VBAC in a subsequent pregnancy, Baranov et al. examined patients with a history of CS with regard to RMT at two specified time points during pregnancy (first vs. second trimester). Both the absolute RMT values and the difference in RMT between the two examinations were determined. The predictive power for TOLAC success of the absolute measured values, as well as of the difference in RMT, was insufficient. In this study, the median RMT was 5.7 mm (IQR 4.0–8.6 mm) in the first trimester and 3.6 mm (IQR 2.2–5.1 mm) in the second trimester; the difference in RMT between the first and second examination was 1.6 mm (IQR 0.2–4.4 mm) [26]. In contrast to the above-mentioned studies, and analogously to our approach, RMT (not LUS) was measured. During the first examination, the uterine scar could be visualized in all patients, allowing reliable RMT measurement; however, during the second examination this was only achieved in 68% of patients. The authors themselves suspected that this was due to the limited discriminative ability of ultrasound to detect pregnancy-related anatomical and histological changes in the scar. In contrast, determining the thickness of the myometrium precisely in the area of the uterotomy is comparatively straightforward after birth, corresponding to our measurement approach. Using TAS, RMT could be determined in 90% (27/30) of our patients, and using TVS in 100% (29/29). Future studies should focus on this area before and after birth to allow a more precise correlation between prepartum and postpartum measurements, e.g., of the HR, which is hypothesized to be independent of LUS stretching.

Naji et al. examined patients who were currently pregnant and had a history of CS, in each trimester, using TVS to measure RMT; measurements were carried out only if the uterotomy could be visualized. The difference in RMT between the first and second trimester examinations was also recorded. The authors showed that the chance of a successful VBAC increases with a larger RMT in the second trimester (OR 6.26 per mm, *p* = 0.0009), and decreases with a greater difference in RMT between the first and second trimester (OR 0.25 per mm, *p* < 0.0001). This suggests that measurements from earlier stages of pregnancy are more predictive of successful TOLAC than measurements taken at more advanced stages. It should be noted that this study was conducted before a standardized definition of uterine niches existed; Naji et al. instead refer to the hypoechogenic part of the scar. The authors postulate that the size of this defect correlates less with the probability of success than the absolute RMT, and that the presence of a pronounced scar defect does not necessarily indicate inadequate wound healing or a compromised scar. This is in line with our approach that the decisive factor is not so much the absolute RMT value but rather the wound healing represented by the HR [34].

Naji et al. further suggest that assessment of the LUS and the uterotomy scar is more informative in the earlier stages of pregnancy than in the (late) third trimester. We believe that assessment of the non-pregnant uterus (in the late follicular phase) may provide even more information about wound healing.

Naji et al. [34] also postulate that the presence of a large uterine defect does not necessarily indicate a defective scar, as long as the RMT is sufficiently thick and remains so during pregnancy. In our cohort, a median RMT of 9.9 mm (IQR 7.5–14.5) was found together with comparatively large defects; on average, these accounted for 50% of the thickness of the total myometrium and could therefore be classified as a large defect. These findings suggest that the presence of a uterine defect can be observed in women with a successful VBAC and therefore does not necessarily preclude successful vaginal delivery. This observation is compatible with the hypothesis that the presence of a uterine defect itself does not necessarily indicate an unstable scar. Our study, however, is not designed to assess the stability of a uterotomy; rather, it merely serves to determine—retrospectively—what degree of residual wall defect remains compatible with a vaginal delivery. It is unlikely that uterine rupture can be fully predicted using ultrasound alone, as the forces exerted and the distribution of tensile stress play a role alongside wall stability.

These findings support the hypothesis that scar healing, rather than the mere presence of a defect, may be relevant for TOLAC success.

In women examined by TVS, the median HR was 0.50 (IQR 0.33–0.66), with the lowest observed value being 0.25. Notably, successful VBAC was observed across this entire range of HR values. However, because this study exclusively included women with successful VBAC and did not include a comparison group of women with failed TOLAC, uterine rupture, or other adverse outcomes, no predictive thresholds can be derived from these findings. Rather, the data provide a descriptive characterization of uterine scar morphology following successful VBAC and may serve as a basis for generating hypotheses regarding potentially relevant sonographic parameters for future studies.

The present findings indicate that uterine defects can frequently be observed among women with successful VBAC and that a defect can involve up to 75% of the wall thickness. These findings can be used in patient counseling to show that the presence of a visible defect does not necessarily imply a worse outcome—though, conversely, such an outcome cannot be ruled out either.

The postpartum uterus undergoes rapid involution, and the sonographic appearance of the uterotomy scar therefore changes dynamically during the first days and weeks after delivery. The present study assessed the scar within the first six days postpartum, in contrast to most previous studies, which conventionally assess uterine niches in the non-pregnant uterus several months after delivery (e.g., at around six months postpartum). Consequently, the sonographic findings observed at time of delivery—including the presence and dimensions of a defect—may not fully correspond to a persistent defect in the non-pregnant uterus once involution and healing are complete.

Spearman correlation analysis revealed no statistically significant association between the day of postpartum examination (within the 1–6-day window) and RMT, defect depth, or healing ratio, for either TAS or TVS (all *p* > 0.05); however, given the small overall sample size and the correspondingly small number of patients examined non-longitudinally, this analysis is likely underpowered to detect a subtle short-term dynamic, and the absence of a significant correlation should therefore not be over-interpreted as evidence against such a dynamic within this early postpartum interval.

In consequence, the study results raise questions: the detected uterine wall defect postpartum in many of the study participants was asymptomatic for the women, at least prior to pregnancy. Known gynecological symptoms of uterine wall defects include postmenstrual spotting and dysmenorrhea, as well as secondary infertility and caesarean scar defects, which represent a recognized risk factor for caesarean scar pregnancy, a rare but potentially severe complication in which the pregnancy implants within the uterine scar [35].

Further studies should investigate the extent to which sonographic findings in the early puerperium correspond to those after completed postpartum healing, and thus whether the present results can be transferred to the non-pregnant uterus. Beyond this, prospective longitudinal studies are needed to determine the long-term clinical relevance of an early postpartum uterine wall defect, including its association with clinical symptoms of caesarean scar disorder and—ideally in studies including both successful and failed TOLAC attempts—the outcome of a subsequent trial of labor. Such studies would help clarify whether the sonographic findings described here have predictive value beyond the descriptive characterization provided in this study.

One limitation of this study is the small number of cases, as this study was conceived as an exploratory pilot investigation for which no comparable prior data were available. Furthermore, the eligible population was inherently small, since only women with a successful VBAC were included and a considerable proportion of women with a prior CS opt for ERCD rather than TOLAC. In addition, other factors such as maternal pre-existing conditions and fetal influences, which can positively or negatively affect the course of birth, were recorded but not considered in the analysis. Factors related to the previous CS surgery technique itself—including surgeon experience, myometrial closure technique, estimated blood loss, and postoperative outcomes—were not systematically recorded and could therefore not be analyzed. As this study focused on the descriptive sonographic characterization of the uterotomy scar rather than its surgical or clinical determinants, these variables were beyond its scope but represent an important direction for future research. In addition, because all sonographic measurements were obtained in consensus by two examiners both present at the same examination intra- or interobserver reproducibility was not explicitly investigated. This study was not designed to predict the success of a TOLAC based on hysterotomy morphology; consequently, there is no control group. The study design is purely descriptive, aiming to demonstrate—for the first time and on a retrospective basis—that even significant uterine wall defects do not preclude a VBAC. Neither predictive cut-off values for successful TOLAC nor uterine dehiscence/rupture can be derived. A limitation of the design is, therefore, that the study cannot contribute to predicting the success of a VBAC.

A strength of this study is that patients were examined using both TAS and TVS, within the first week after birth, reflecting the actual constellation of the uterine wall at the uterotomy site in the immediate postpartum period. However, inaccuracies within the 6-day range cannot be ruled out due to the lack of longitudinal measurements.

## 5. Conclusions

In summary, within the first few days postpartum a sonographic uterotomy defect of ≥2 mm could be visualized in 82.8% and covered at least 50% of the wall height in half of the subjects, shown by a median HR of 0.5 after uncomplicated VBAC. Rather, our findings simply suggest that a sonographic uterine wall defect detected in the early postpartum period is compatible with a preceding successful VBAC and should therefore not, by itself, be regarded as evidence of insufficient scar healing.

As this study exclusively included women with a successful VBAC and did not include a comparison group of women with failed TOLAC or uterine rupture, these findings cannot be used to establish predictive cut-off values or to determine the clinical safety of specific defect dimensions.

The HR may represent a potentially useful descriptive parameter for characterizing scar morphology, independently of the total myometrial thickness, which varies between pregnancy, gestational age and non-pregnant state. However, its predictive value for TOLAC outcome remains to be established in future prospective, controlled studies including women with both successful and failed TOLAC.

## Figures and Tables

**Table 1:** Patients’ demographic and sonographic characteristics at time of examination.

Parameter	N	Mean (±STD)	Median (IQR)
Time after VBAC [days]	30	2.1 (1.0)	2.0 (1.0–2.0)
Age [years]	30	32.5 (5.3)	33.5 (30.0–36.0)
BMI [kg/m^2^]	30	29.4 (5.8)	28.0 (25.3–31.8)
RMT using TAS [mm]	27	14.0 (7.0)	12.0 (8.0–19.0)
RMT using TVS [mm]	29	11.1 (5.0)	9.9 (7.5–14.5)
Defect depth using TAS [mm]	17	11.4 (3.9)	12.2 (7.7–14.8)
Defect depth using TVS [mm]	24	10.6 (4.8)	9.9 (6.5–15.6)
Healing ratio using TAS	17	0.50 (0.17)	0.55 (0.30–0.62)
Healing ratio using TVS	24	0.51 (0.19)	0.50 (0.33–0.66)

Abbreviations: BMI, body mass index; IQR, interquartile range; RMT, residual myometrial thickness; STD, standard deviation; TAS, transabdominal sonography; TVS, transvaginal sonography; VBAC, vaginal birth after caesarean section.

## Data Availability

The data presented in this study are available on request from the corresponding author due to privacy and ethical restrictions.

## Abbreviations

BMI	Body mass index
CS	Caesarean section
ERCD	Elective repeat caesarean delivery
HR	Healing ratio
HSG	Hysterosonography
IQR	Interquartile range
LUS	Lower uterine segment
PPH	Peripartum hemorrhage
RMT	Residual myometrial thickness
STD	Standard deviation
TAS	Transabdominal sonography
TOLAC	Trial of labor after caesarean delivery
TVS	Transvaginal sonography
VBAC	Vaginal birth after caesarean section
